# Improved Lipophilicity Is Associated with the Cytotoxic Activity of Chlorogenic Acid Esters in In Vitro Colorectal Cancer Models

**DOI:** 10.3390/molecules31172952

**Published:** 2026-08-23

**Authors:** Ana María Castañeda-Cifuentes, Johanna Pedroza-Díaz, Gloria A. Santa-González, Isabel Cristina Henao-Castañeda, Andrea Johanna Andrea Báez, Jorge L. Jios, Ana Laura Di Virgilio

**Affiliations:** 1 de Investigación e Innovación Biomédica GI^2^B, Facultad de Ciencias Exactas y Aplicadas, Institución Universitaria ITM, Medellín 050012, Colombia; anacastaneda225721@correo.itm.edu.co (A.M.C.-C.); gloriasanta@itm.edu.co (G.A.S.-G.); 2 Grupo de Investigación en Biología Médica (BioMed), Facultad de Ciencias Exactas y Aplicadas, Institución Universitaria ITM, Medellín 050012, Colombia; 3 Grupo de Investigación en Sustancias Bioactivas, Facultad de Ciencias Farmacéuticas y Alimentarias, Universidad de Antioquia, Medellín 050010, Colombia; isabel.henao@udea.edu.co (I.C.H.-C.); andrea.baez@udea.edu.co (A.J.A.B.); 4 Laboratorio UPL (UNLP-CIC), Camino Centenario e/505 y 508 (1897) M.B. Gonnet, Departamento de Química, Facultad de Ciencias Exactas, Universidad Nacional de La Plata, República Argentina, 47 esq. 115, La Plata 1900, Buenos Aires, Argentina; jljios@quimica.unlp.edu.ar; 5 Centro de Química Inorgánica (CEQUINOR, CONICET), Facultad de Ciencias Exactas, Universidad Nacional de la Plata Facultad, 47 Y 115, La Plata 1900, Buenos Aires, Argentina; aldivirgilio@biol.unlp.edu.ar

**Keywords:** *n*-butyl chlorogenate, colorectal cancer, chlorogenic acid, esterification, oxidative stress, cell death, chlorogenic acid esters

## Abstract

Chlorogenic acid (CGA) exhibits anticancer activity in colorectal cancer (CRC), but its clinical application is limited by low lipophilicity. To improve its physicochemical properties, four CGA esters (methyl, ethyl, *n*-propyl, and *n*-butyl chlorogenates) were synthesized and evaluated. Physicochemical properties were characterized in silico, and their biological activity was assessed in SW480, HT-29, and non-tumoral NCM460 cell lines using viability assays and flow cytometry. Molecular docking studies were performed to investigate the interactions of CGA and its esters with proteins involved in cell-proliferation-related signaling pathways. In silico analysis showed a progressive increase in LogP values across ester derivatives. All esters complied with Lipinski’s rule of five, whereas none met Veber’s rule due to their predicted topological polar surface area (TPSA) values. The esters induced dose- and time-dependent reductions in cell viability, with *n*-butyl chlorogenate exhibiting the strongest cytotoxic activity and a significantly lower IC_50_ value within the tested concentration range. This derivative showed preferential cytotoxic activity toward SW480 cells while exhibiting only limited effects in non-tumoral NCM460 cells. In addition, *n*-butyl chlorogenate induced changes in mitochondrial oxidative status and phosphatidylserine externalization, consistent with apoptosis-associated cellular changes. Overall, these findings demonstrate that esterification modifies the physicochemical profile of CGA ester derivatives and is associated with enhanced cytotoxic activity. Increased lipophilicity was associated with enhanced cytotoxic activity, supporting further optimization of these compounds for CRC research.

## 1. Introduction

Colorectal cancer (CRC) is the fourth most frequently diagnosed cancer worldwide and the fourth leading cause of cancer-related mortality. Its incidence is higher in developed countries and occurs more often in men than in women [1]. Globally, CRC incidence is projected to increase by 70.5% by 2045, while CRC-related mortality is expected to rise by 83.4% over the same period [2]. Given these projections and the disease’s high mortality rate, there is an urgent need to develop more effective preventive and therapeutic strategies. Current treatment approaches, including radiotherapy and chemotherapy, rely on cytotoxic agents that are associated with significant adverse effects and exhibit limited specificity and therapeutic efficacy [3,4,5]. More than 94% of CRC cases have at least one altered protein in the Wnt/β-catenin signaling pathway due to genetic and epigenetic mechanisms [6].

Among potential chemopreventive agents, natural compounds have attracted considerable interest due to their favorable safety profiles and long-term dietary exposure. Chlorogenic acid (CGA), one of the most abundant dietary phenolic compounds, is widely distributed in coffee, tea, fruits, and vegetables [7]. Epidemiological and experimental studies have suggested that CGA may contribute to CRC chemoprevention by modulating oxidative stress, inflammation, cell proliferation, and apoptosis [8]. In addition, in vitro studies have demonstrated that CGA exerts cytotoxic effects against CRC cells [9].

Despite these promising biological properties, the application of CGA remains limited by its low lipophilicity, which has been associated with reduced cellular uptake [10]. Esterification of natural compounds has been shown to modify their physicochemical and pharmacokinetic properties, potentially enhancing their biological activity, as reported for caffeic acid [11,12,13] and ferulic acid [14]. Likewise, structural modifications of the carboxylic acid moiety of CGA have been shown to preserve its biological properties while improving characteristics relevant to drug development, including stability and predicted pharmacokinetic behavior [15,16].

Altogether, the available evidence suggests that esterification represents a promising strategy for optimizing the biological properties of natural compounds. However, the effects of esterification of the carboxylic acid moiety of CGA on CRC cells remain poorly understood. Therefore, the present study evaluated a series of CGA ester derivatives in two widely used CRC cell lines, thereby assessing their cytotoxic activity across different cellular contexts.

The CRC model selected for this research consists of two cell lines, SW480 and HT-29, with different truncating mutations in the APC gene [17,18,19], which result in different levels of transcriptional activity of the Wnt/β-catenin signaling pathway. This pathway plays a critical role in the development and progression of colorectal cancer. Additionally, the non-tumoral human colon cell line NCM460 was included as a control.

Moreover, polyphenols such as CGA have been reported to modulate the Wnt/β-catenin pathway. A previous study identified in silico interactions between CGA and β-catenin and LRP6, suggesting potential modulation of the pathway and providing a starting point for understanding CGA’s effect in the context of CRC [20]. Moreover, in a Top/Fop Flash assay, a greater decrease in transcriptional activation of the Wnt/β-catenin pathway was observed with CGA compared with the specific inhibitor iCRT14 in the SW480 cell line [9].

Additionally, a qPCR analysis showed changes in mRNA expression levels of the CTNNB1 (β-catenin) and CCND1(Cyclin D1) genes. The β-catenin mRNA levels in both SW480 and HT-29 cells were negatively regulated. Likewise, Cyclin D1 expression levels decreased significantly, as expected, while Cyclin D1 is a target in the Wnt/β-catenin pathway [9].

The aim of this study is to improve the cytotoxic effect of CGA in an in vitro CRC model through structural modifications; therefore, we hypothesized that esterification of the carboxylic acid group would modify its physicochemical properties and enhance the cytotoxic activity against CRC cells.

## 2. Results

### 2.1. Validation of the Structural Modification on the CGA

The nuclear magnetic resonance (NMR) spectra of the synthesized CGA esters are provided in the Supplementary Materials (Figures S1–S4). The synthesis process prioritized the obtention of high-purity material for subsequent structural characterization and biological evaluation. The profiles of the CGA esters are summarized below:

Methyl chlorogenate: RT 13.8 min ^1^H NMR (600 MHz, CD_3_OD) δ 7.53 (d, J = 16 Hz, 1H), 7.04 (t, J = 2 Hz, 1H), 6.95 (dt, J = 8 and 2 Hz, 1H), 6.79 (dd, J = 8 and 1 Hz, 1H), 6.22 (dd, J = 16 and 1 Hz, 1H), 5.28 (td, J = 8 and 4 Hz, 1H), 4.14 (dt, J = 7 and 3 Hz, 1H), 3.73 (dd, J = 8 and 3 Hz, 1H), 3.70 (d, J = 1 Hz, 3H), 2.24–2.11 (m, 3H), 2.01 (dd, J = 14 and 7 Hz, 1H) ppm.

Ethyl chlorogenate: RT 15.01 min ^1^H NMR (600 MHz, CD_3_OD) δ 7.53 (d, J = 16 Hz, 1H), 7.04 (d, J = 2 Hz, 1H), 6.94 (dd, J = 8 and 2 Hz, 1H), 6.78 (d, J = 8 Hz, 1H), 6.22 (d, J = 16 Hz, 1H), 5.28 (td, J = 8 and 5 Hz, 1H), 4.22–4.07 (m, 3H), 3.73 (dd, J = 8 and 3 Hz, 1H), 2.24–2.11 (m, 3H), 2.01 (dd, J = 14 and 7 Hz, 1H), 1.24 (t, J = 7 Hz, 3H) ppm.

*n*-propyl chlorogenate: RT 16.2 min ^1^H NMR (600 MHz, CD_3_OD) δ 7.52 (d, J = 16 Hz, 1H), 7.04 (d, J = 2 Hz, 1H), 6.95 (dd, J = 8 and 2 Hz, 1H), 6.78 (d, J = 8 Hz, 1H), 6.21 (d, J = 16 Hz, 1H), 5.31–5.25 (m, 1H), 4.14 (dt, J = 7 and 4 Hz, 1H), 4.04 (ddt, J = 32, 11 and 7 Hz, 2H), 3.73 (dd, J = 7 and 3 Hz, 1H), 2.25–2.12 (m, 3H), 2.01 (dd, J = 4 and 7 Hz, 1H), 1.71–1.59 (m, 2H), 0.91 (t, J = 7 Hz, 3H) ppm.

*n*-butyl chlorogenate: RT 17.4 min. ^1^H NMR (600 MHz, CD_3_OD) δ 7.43 (d, J = 16 Hz, 1H), 6.95 (d, J = 2 Hz, 1H), 6.85 (dd, J = 8 and 2 Hz, 1H), 6.70 (d, J = 8 Hz, 1H), 6.12 (d, J = 16 Hz, 1H), 5.21–5.14 (m, 1H), 4.07–3.93 (m, 3H), 3.65 (dd, J = 7 and 3 Hz, 1H), 2.15–2.03 (m, 3H), 1.90 (dd, J = 14 and 7 Hz, 1H), 1.56–1.44 (m, 2H), 1.30–1.19 (m, 2H), 0.79 (t, J = 7 Hz, 3H) ppm. ^13^C NMR (151 MHz, CD_3_OD) δ 173.6 (8), 166.9 (15), 148.2 (25), 145.8 (24), 145.4 (21), 126.1 (22), 121.5 (27), 115.1 (26), 113.7 (20), 113.5 (23), 74.2 (1), 70.7 (3; 4), 68.70 (5), 64.95 (10), 35.2 (2;6), 30.1 (11), 18.6 (12), 12.5 (13) ppm.

### 2.2. In Silico Studies

#### 2.2.1. In Silico Physicochemical Characterization

In silico physicochemical characterization evaluated Lipinski’s rule of five as an indicator of drug-like properties (molecular weight < 500 Da, calculated LogP < 5, fewer than 10 hydrogen-bond acceptors, and fewer than 5 hydrogen-bond donors) [21]. In addition, Veber’s rule (topological polar surface area ≤ 140 ${Å}^{2}$ and rotatable bonds ≤ 10) was evaluated as a complementary descriptor of drug-like properties. Lipophilicity and aqueous solubility were further characterized using predicted Consensus LogP and LogS (ESOL) values, respectively.

Based on these analyses, CGA and its ester derivatives exhibited distinct physicochemical profiles. All CGA esters complied with Lipinski’s rules, although native CGA presented one violation due to its high hydrogen-bond donor count. Nevertheless, esterification produced a small increase in Consensus LogP with each added carbon, accompanied by a decrease in predicted aqueous solubility (LogS), indicating that alkyl chain length modified the physicochemical properties of the ester derivatives (Table 1).

These in silico findings provided a physicochemical characterization of the synthesized esters and served as complementary information for the subsequent in vitro evaluation.

#### 2.2.2. Molecular Docking of CGA and Ester Derivatives with β-Catenin Subsites

Molecular docking studies were performed in the two reported β-catenin subsites using parameters reported for the study of β-catenin interactions with CGA and the validation compounds C2 and iCRT14 for the allosteric and union sites, respectively [20]. CGA showed higher affinity than the ester derivatives for both β-catenin subsites. Results are shown in Table 2.

The conformation of the ester derivatives at the β-catenin union site was similar to that of CGA, except for ethyl chlorogenate, which differed in the apolar tail orientation. In the Allosteric site, the conformation of methyl chlorogenate is equivalent to that of CGA, and the other esters interact with β-catenin in a similar conformation, but with a displacement relative to CGA.

At the β-catenin union site, CGA showed the strongest binding energy (−6.5 kcal/mol), followed by methyl and ethyl chlorogenate (−6.3 kcal/mol each), *n*-propyl chlorogenate (−6.0 kcal/mol), and *n*-butyl chlorogenate (−5.6 kcal/mol). This progressive decrease in binding affinity correlated with a reduction in the number of hydrogen bonds, from six in CGA and ethyl chlorogenate to five in methyl chlorogenate and only three in the *n*-propyl and *n*-butyl derivatives. Furthermore, CGA, methyl, and ethyl chlorogenate formed an additional π-stacking interaction with HIS470, which probably contributed to their stronger binding energies relative to the *n*-propyl and *n*-butyl derivatives.

**Figure 1:**
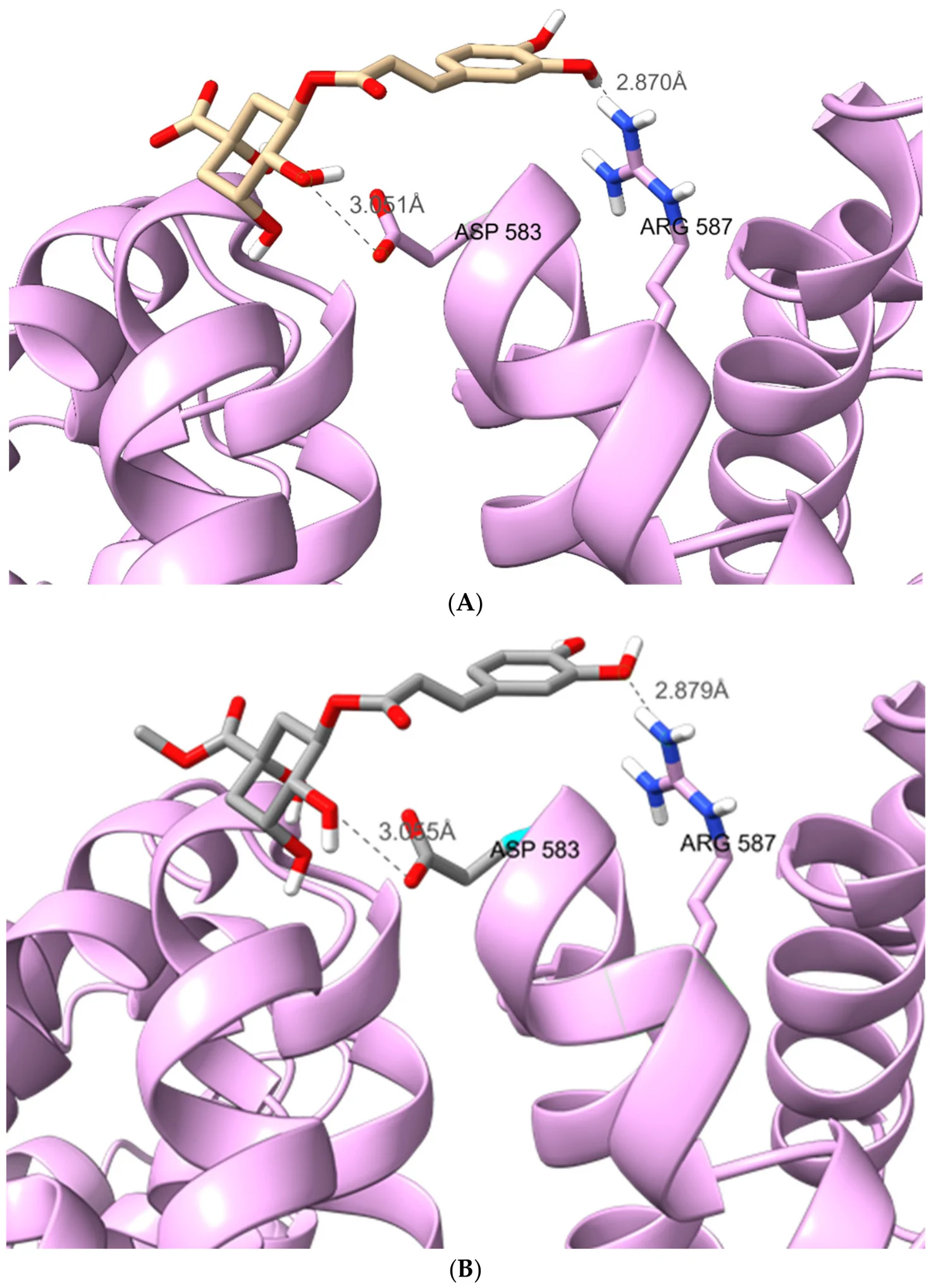
(**A**) CGA (beige) interaction with the β-catenin allosteric site (pink) with critical hydrogen-bond distances. (**B**) Methyl chlorogenate (gray) interaction with the β-catenin allosteric site (pink) with critical hydrogen-bond distances.

At β-catenin, CGA exhibited a binding energy of −5.5 kcal/mol and formed hydrogen bonds with ASP583 (3.051 Å) and ARG587 (2.870 Å). Methyl chlorogenate closely mimicked this interaction with a binding energy of −5.4 kcal/mol and a hydrogen bond to ASP583 at 3.055 Å. In contrast, ethyl, *n*-propyl, and *n*-butyl chlorogenates showed slightly lower binding energies of −5.0 kcal/mol, with hydrogen bond distances to ASP583 ranging from 3.481 to 3.500 Å and to ARG587 from 2.833 to 2.964 Å, highlighting their comparable binding profiles (Figure 1A,B).

### 2.3. Chlorogenic Acid Esters Affect the Viability of SW480 and HT-29 Colorectal Cancer Cells

**Figure 2:**
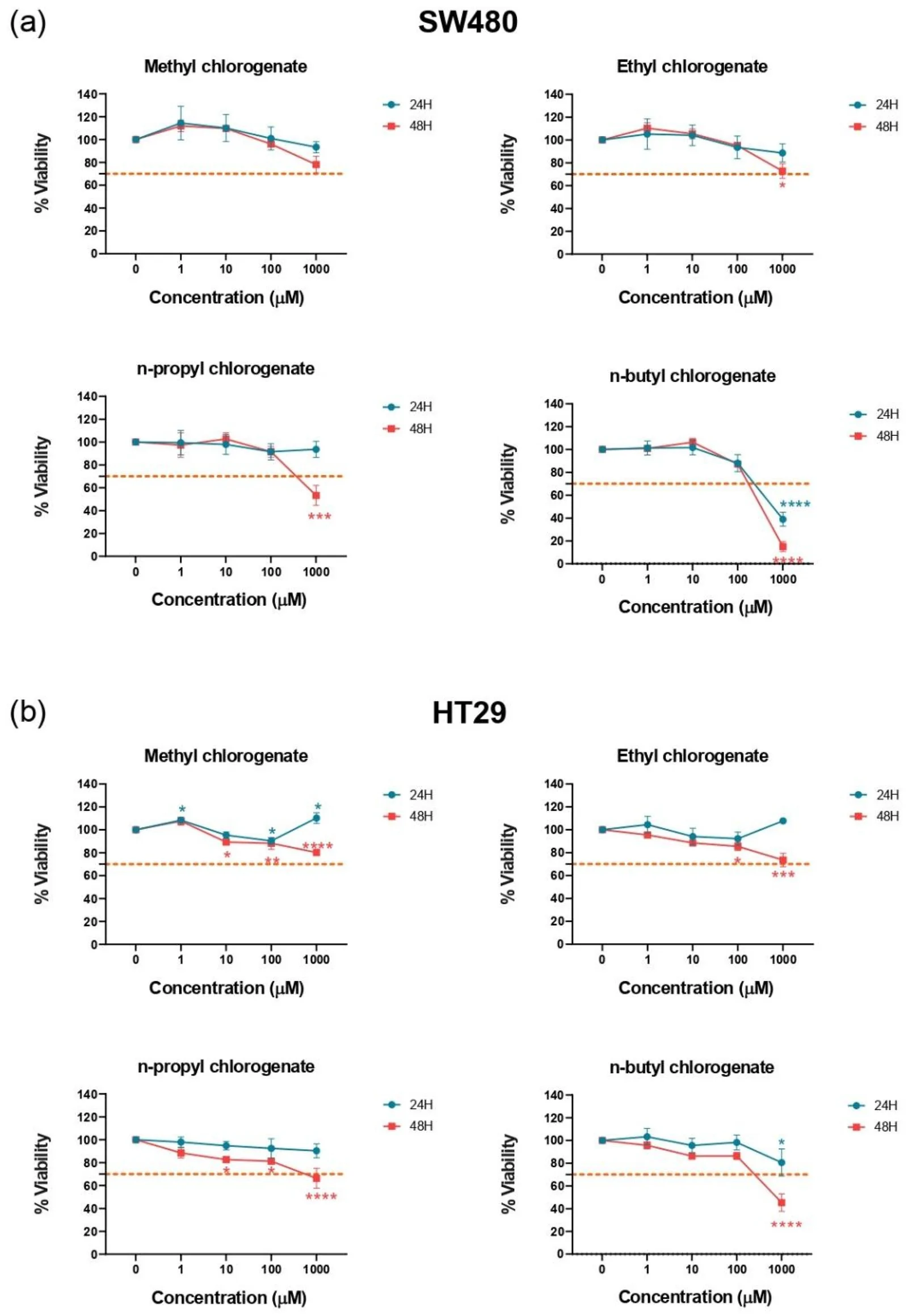
Dose–response curves of CGA esters in CRC cell lines. Cells were exposed to 0, 1, 10, 100, or 1000 μM CGA esters for 24 or 48 h. The dashed line represents the cytotoxicity threshold established by ISO 10993-5:2009. (**a**) Viability of SW480 cells and (**b**) HT-29 cells. Differences were considered statistically significant at *p* < 0.05. Asterisks indicate significant differences relative to the corresponding control: *p* < 0.05 (*), *p* < 0.01 (**), *p* < 0.001 (***), and *p* < 0.0001 (****).

Exposure of SW480 and HT-29 cells to CGA esters for 24 h and 48 h significantly reduced cell viability at concentrations ≥ 100 µM (*p* < 0.05) compared with the untreated control (0 µM), indicating a dose-dependent cytotoxic effect for all CGA esters (Figure 2). For comparison, the IC_50_ of native CGA in these cell lines was previously evaluated by our group and reported in previous studies [9,20].

This reduction became more pronounced after 48 h, demonstrating a time-dependent response in both cell lines. SW480 cells were more sensitive than HT-29 cells to *n*-propyl and *n*-butyl chlorogenates, whereas methyl and ethyl chlorogenate produced comparable effects in both cell lines.

**Figure 3:**
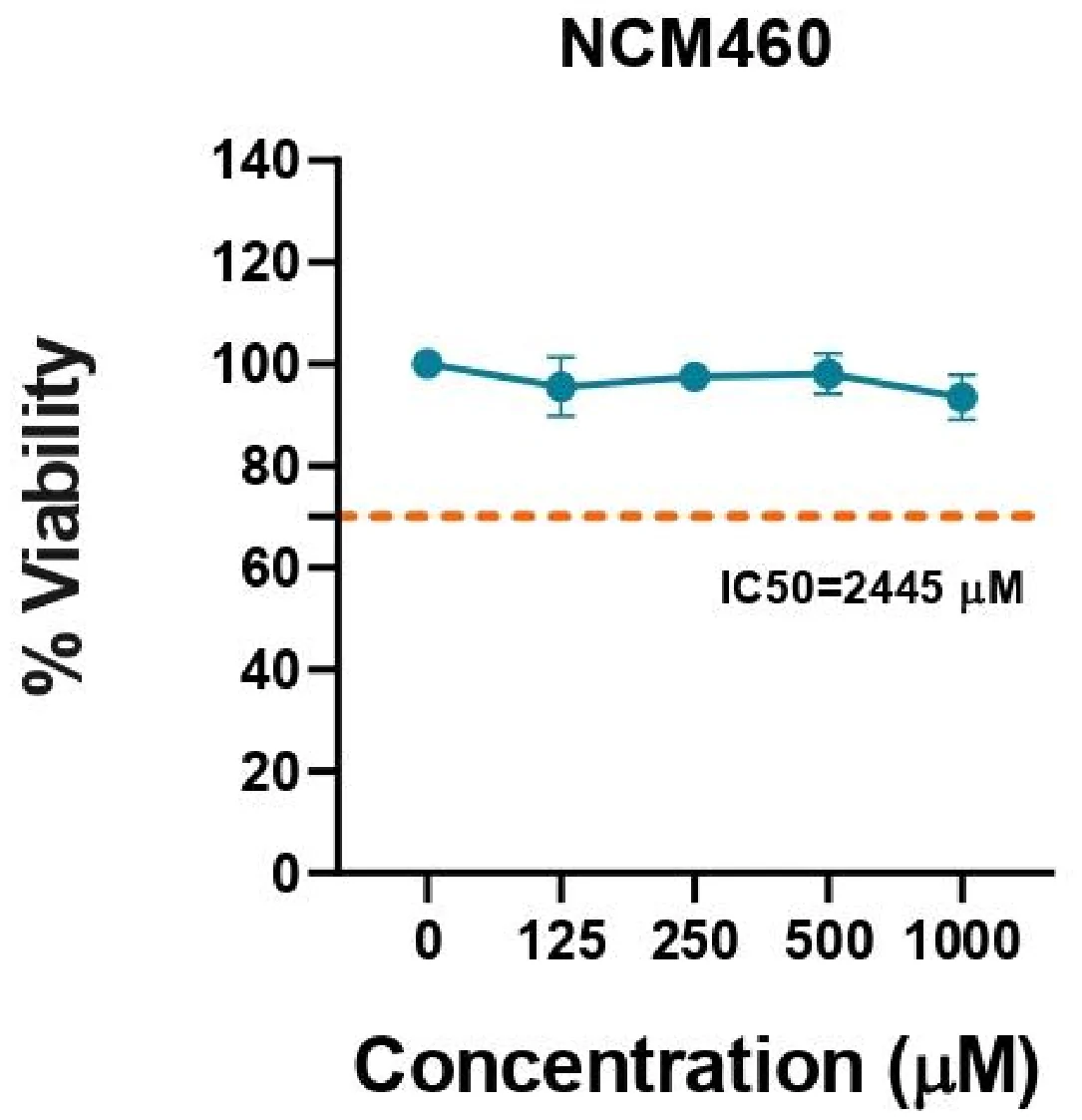
Viability of NCM460 normal human colon cell line after 24 h exposure to *n*-butyl chlorogenate.

Table 3 presents the half-maximal inhibitory concentration (IC_50_) values of the evaluated CGA esters. IC_50_ values were obtained by nonlinear regression of the dose–response curves. For some compounds, the calculated IC_50_ values exceeded the highest concentration tested (1000 µM) and therefore represent model-derived estimates. Progressive addition of carbons to the CGA ester derivatives showed an inverse-proportional IC_50_ value in both cell lines and over time. The greatest reduction was observed with *n*-butyl chlorogenate in the SW480 cell line. The orange line in Figure 3 represents the cytotoxicity threshold established by ISO 10993-5 [22] for evaluating in vitro evaluation of medical devices, according to which cell viability below 70% is considered cytotoxic. Consistent with this criterion, *n*-propyl and *n*-butyl chlorogenates produced cytotoxic effects in both cell lines at 1000 µM.

Owing to its favorable cytotoxic profile and low IC_50_ values, *n*-butyl chlorogenate was selected for subsequent experiments. Mechanistic assays were performed after 24 h of exposure, because the cytotoxic effect at 1000 μM was less pronounced than after 48 h.

### 2.4. Differential Cytotoxic Effects of n-Butyl Chlorogenate in Colorectal Cancer Cell Lines

The cytotoxic effect of *n*-butyl chlorogenate was evaluated in the non-tumoral human colon cell line NCM460 and in the CRC cell lines SW480 and HT-29 after 24 h of exposure. Cell viability assays revealed a greater reduction in SW480 cells than in HT-29 and NCM460 cells, with a statistically significant decrease relative to the control observed at 1000 µM (*p* < 0.05) (Figure 3). In contrast, HT-29 cells showed no significant reduction in viability within the tested concentration range. IC_50_ values further supported this observation, with *n*-butyl chlorogenate showing a lower IC_50_ in SW480 cells than in HT-29 cells (Table 3). In contrast, the calculated IC_50_ in NCM460 cells was 2445 µM. IC_50_ values were obtained by nonlinear regression of the dose–response curves. For some compounds, the calculated IC_50_ values exceeded the highest concentration tested (1000 µM) and therefore represent model-derived estimates (Figure 3), indicating lower sensitivity of non-tumoral cells to the compound. Accordingly, *n*-butyl chlorogenate exhibited preferential cytotoxicity toward CRC cells, particularly SW480 cells, while limited effects were observed in NCM460 cells within the tested concentration range.

Given the reduction in CRC cell viability, subsequent analyses were conducted to determine whether these effects were associated with alterations in membrane integrity and cellular morphology.

### 2.5. n-Butyl Chlorogenate Affects the Cell Membrane in SW480 and HT-29 Colorectal Cancer Cells

To further determine whether the reduction in cell viability was associated with alterations in membrane integrity and cellular morphology, flow cytometry was performed using propidium iodide (PI) staining. PI is a membrane-impermeable dye that intercalates into DNA and is commonly used to assess loss of plasma membrane integrity. In addition, forward scatter (FSC) and side scatter (SSC) parameters were analyzed to evaluate changes in cell size and internal complexity, respectively.

**Figure 4:**
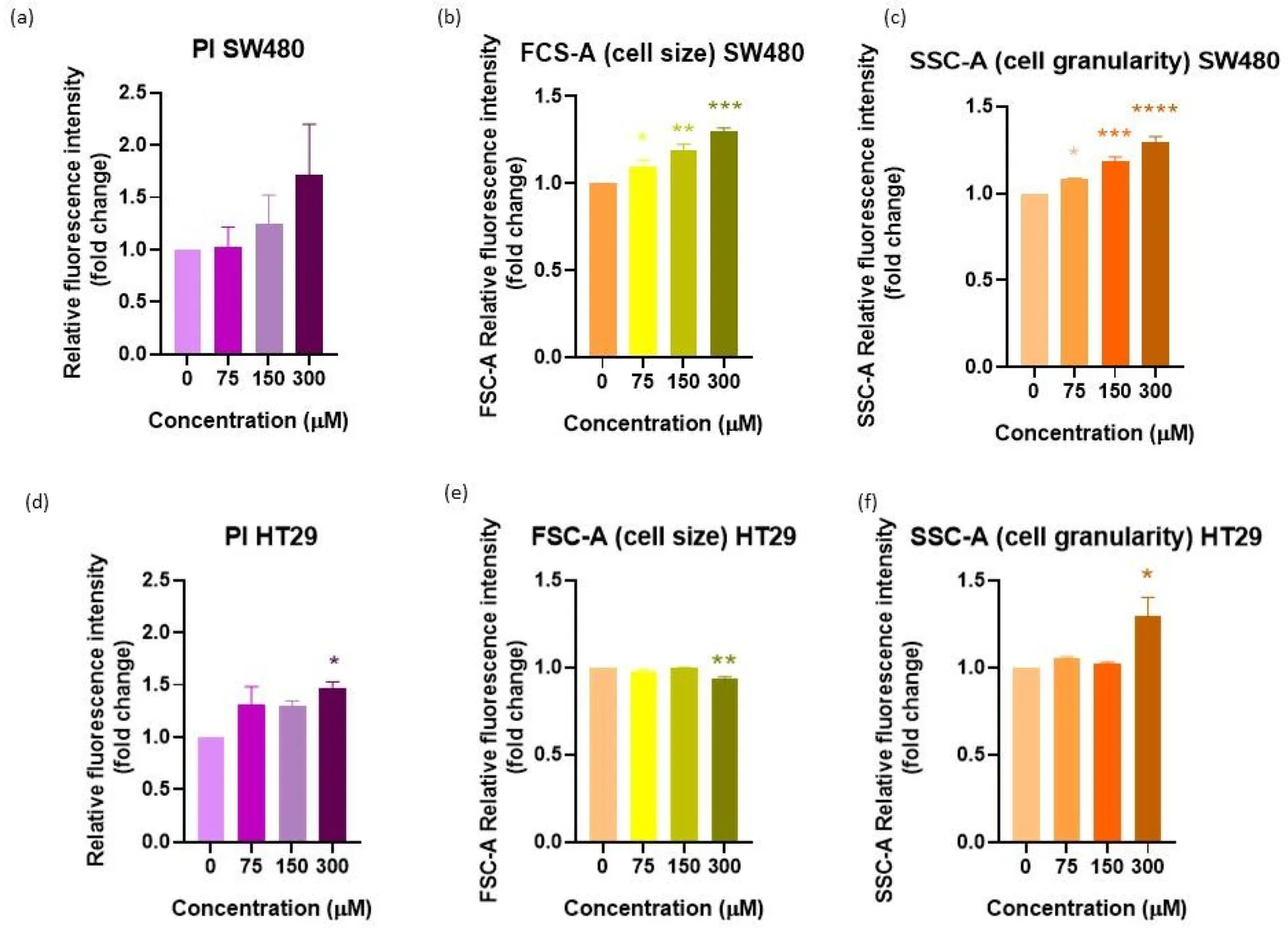
Flow cytometry analysis of propidium iodide (PI) staining and cell size (FSC) and granularity (SSC) in CRC cell lines exposed to *n*-butyl chlorogenate. Quantitative bar graphs show (**a**) PI positivity, (**b**) FSC, and (**c**) SSC in SW480 cells, and (**d**) PI positivity, (**e**) FSC, and (**f**) SSC in HT-29 cells. PI staining was used to evaluate membrane integrity, whereas FSC and SSC parameters were analyzed to assess changes in cell size and internal complexity, respectively. Data are expressed as mean ± SEM from three independent experiments. Differences were considered statistically significant at *p* < 0.05. Asterisks indicate significant differences relative to the corresponding control: *p* < 0.05 (*), *p* < 0.01 (**), *p* < 0.001 (***), *p* < 0.0001 (****).

Exposure to *n*-butyl chlorogenate at 0, 75, 150, and 300 µM resulted in an increasing proportion of PI-positive SW480 and HT-29 cells, indicating alterations in membrane integrity (Figure 4). Treated SW480 cells also exhibited dose-dependent increases in cell size (FSC) and cell granularity (SSC) (Figure 4b,c), suggesting modifications in cellular morphology and internal complexity. These alterations were less pronounced in HT-29 cells. At 300 µM, however, a significant decrease in cell size and a concurrent increase in granularity were observed, suggesting both internal and external cellular alterations associated with cell death. (Figure 4e,f).

### 2.6. Mitochondrial Alterations in SW480 and HT-29 Colorectal Cancer Cells Following n-Butyl Chlorogenate Exposure

Given the alterations in membrane integrity detected by PI staining, mitochondrial function was further evaluated following treatment with *n*-butyl chlorogenate. Changes in mitochondrial membrane potential (ΔΨm) were assessed using DiOC, a membrane potential-dependent fluorescent probe, while MitoTracker Red CM-H2Xros, a ROS-sensitive Mitochondrial probe, was used to indirectly assess changes in mitochondrial oxidative status, as this probe becomes fluorescent upon oxidation and accumulates in mitochondria under oxidative conditions.

**Figure 5:**
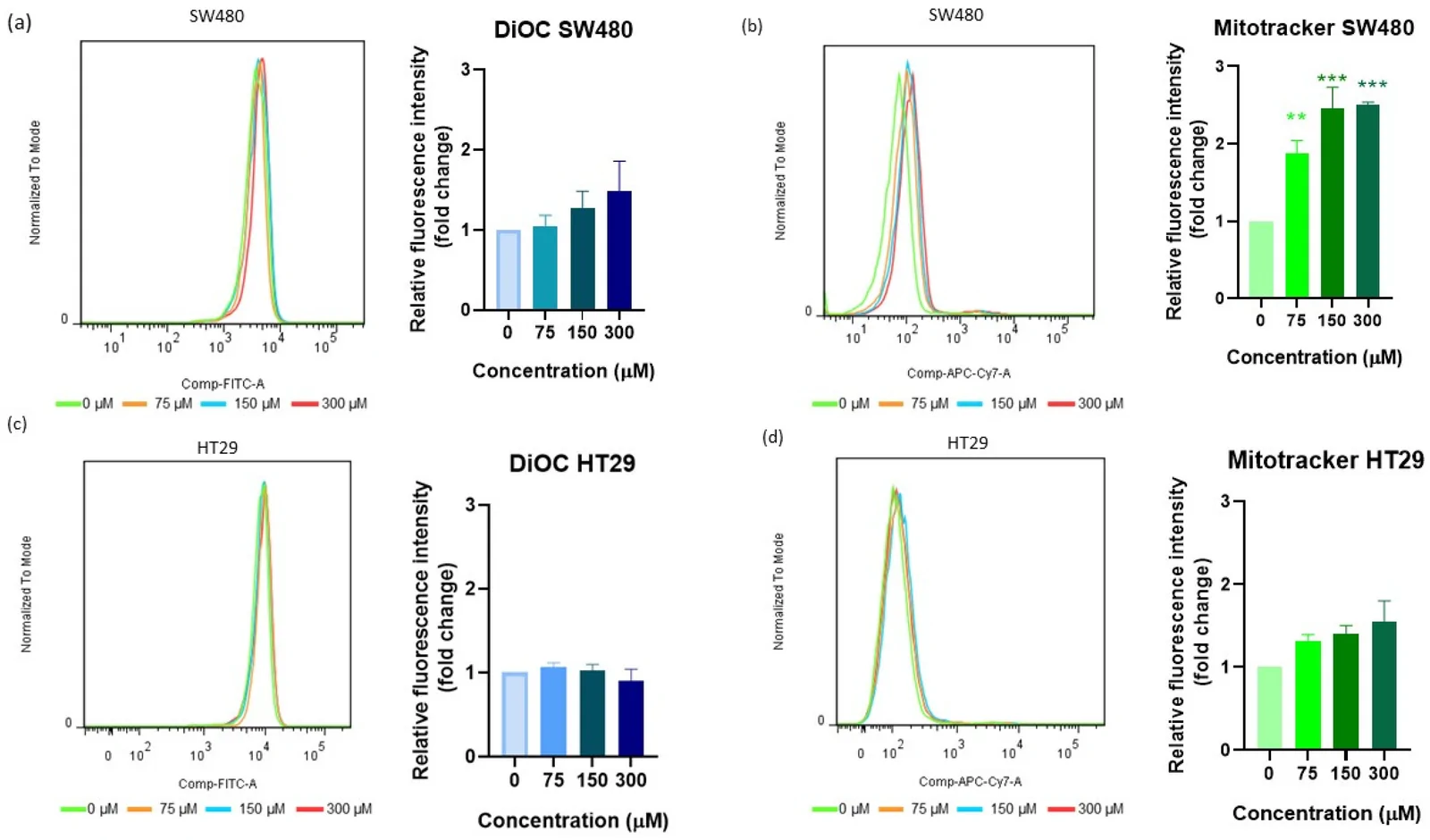
Representative histograms and quantitative flow cytometry analysis using (**a**,**c**) DiOC fluorescence to assess mitochondrial membrane potential; (**b**,**d**) MitoTracker Red CM-H2Xros fluorescence was used as an indirect indicator of changes in mitochondrial oxidative status. Data are expressed as mean ± SEM from three independent experiments. Differences were considered statistically significant at *p* < 0.05. Asterisks indicate significant differences relative to the corresponding control: *p* < 0.01 (**) and *p* < 0.001 (***).

Histograms and quantitative analysis of DiOC fluorescence revealed no significant changes in ΔΨm in either SW480 or HT-29 cells compared with the untreated control (0 µM) (Figure 5a,c). In contrast, MitoTracker fluorescence showed a significant, dose-dependent increase in SW480 cells, whereas HT-29 cells exhibited only a slight, non-significant increase (Figure 5b,d).

Together, these results indicate that, under the conditions evaluated, *n*-Butyl chlorogenate did not induce detectable mitochondrial depolarization but was associated with increased mitochondrial oxidative activity in SW480 cells.

### 2.7. Effects of n-Butyl Chlorogenate on Apoptosis-Associated Markers in SW480 and HT-29 Colorectal Cancer Cells

Following assessment of mitochondrial parameters, additional analyses were performed to determine whether the observed cellular alterations were associated with apoptosis-related cellular changes. Annexin V staining was used to detect the externalization of phosphatidylserine (PS) to the outer leaflet of the plasma membrane, a feature commonly associated with early apoptosis. Caspase-3/7 activation was also analyzed as an indicator of effector caspase involvement in apoptotic signaling.

**Figure 6:**
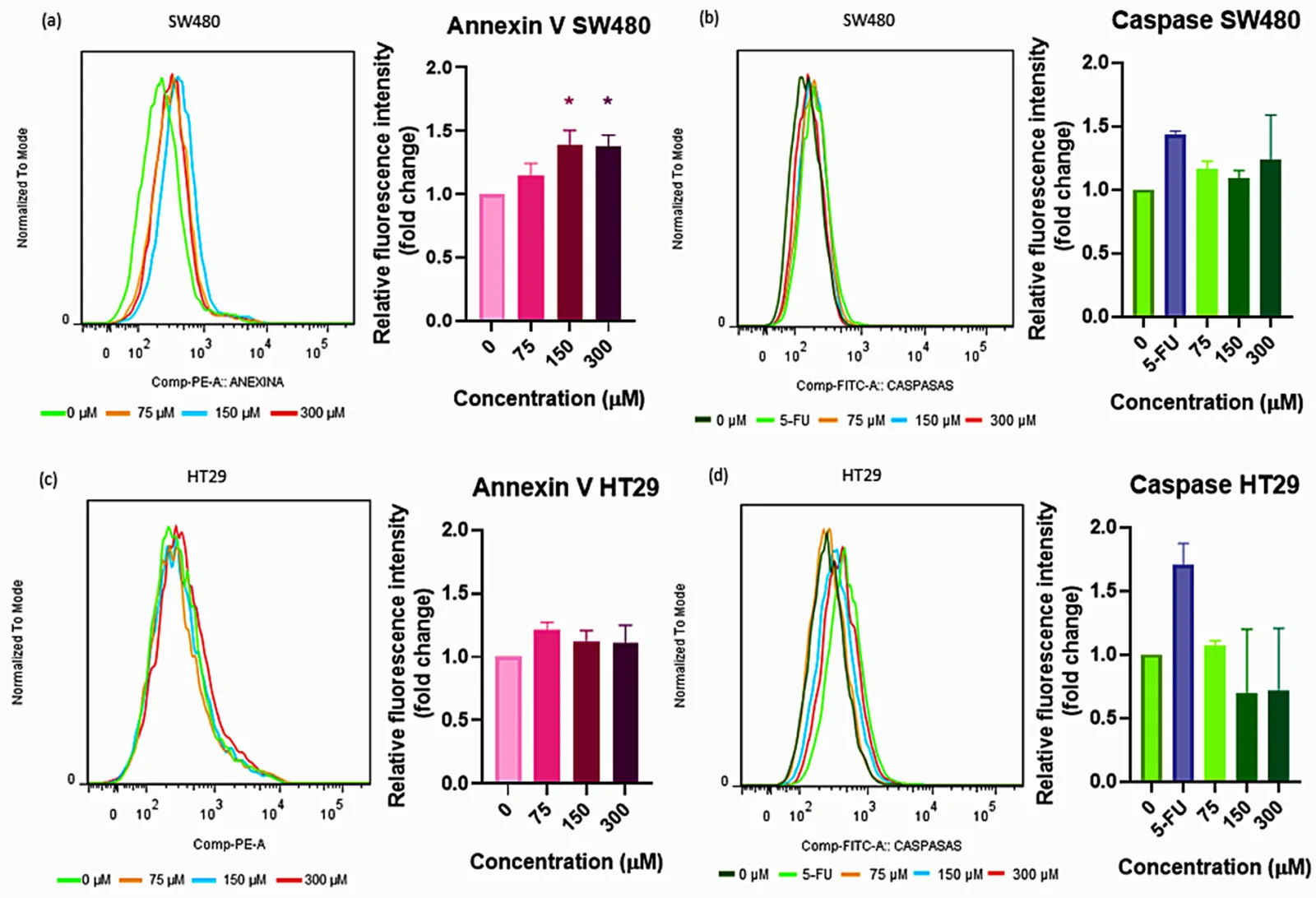
Flow cytometry analysis of apoptosis-associated markers in CRC cell lines exposed to *n*-butyl chlorogenate. (**a**,**c**) Annexin V staining was used to detect phosphatidylserine (PS) externalization, a feature commonly associated with early apoptosis. (**b**,**d**) Caspase-3/7 activation was assessed as a marker of effector caspase activity. Representative histograms and corresponding quantitative analyses are shown for SW480 and HT-29 cells exposed to 0, 75, 150, and 300 µM *n*-butyl chlorogenate. Data are presented as mean ± SEM from three independent experiments. Differences were considered statistically significant at *p* < 0.05. Asterisks indicate significant differences compared to the corresponding control: *p* < 0.05 (*).

Representative histograms and quantitative analysis of Annexin V staining revealed a significant increase in PS externalization in SW480 cells following exposure to *n*-butyl chlorogenate at 75,150, and 300 µM compared with the untreated control (0 µM). In contrast, HT-29 cells exhibited no significant changes under the same exposure conditions (Figure 6a,c). However, Caspase-3/7 activation did not differ significantly from the control in either SW480 or HT-29 at any of the concentrations tested (Figure 6b,d). These findings indicate that *n*-butyl chlorogenate induced phosphatidylserine externalization in SW480 cells without detectable activation of Caspase-3/7 at the evaluated time point.

### 2.8. Effect of the n-Butyl Chlorogenate on Cell Proliferation in SW480 and HT-29 Colorectal Cancer Cells

**Figure 7:**
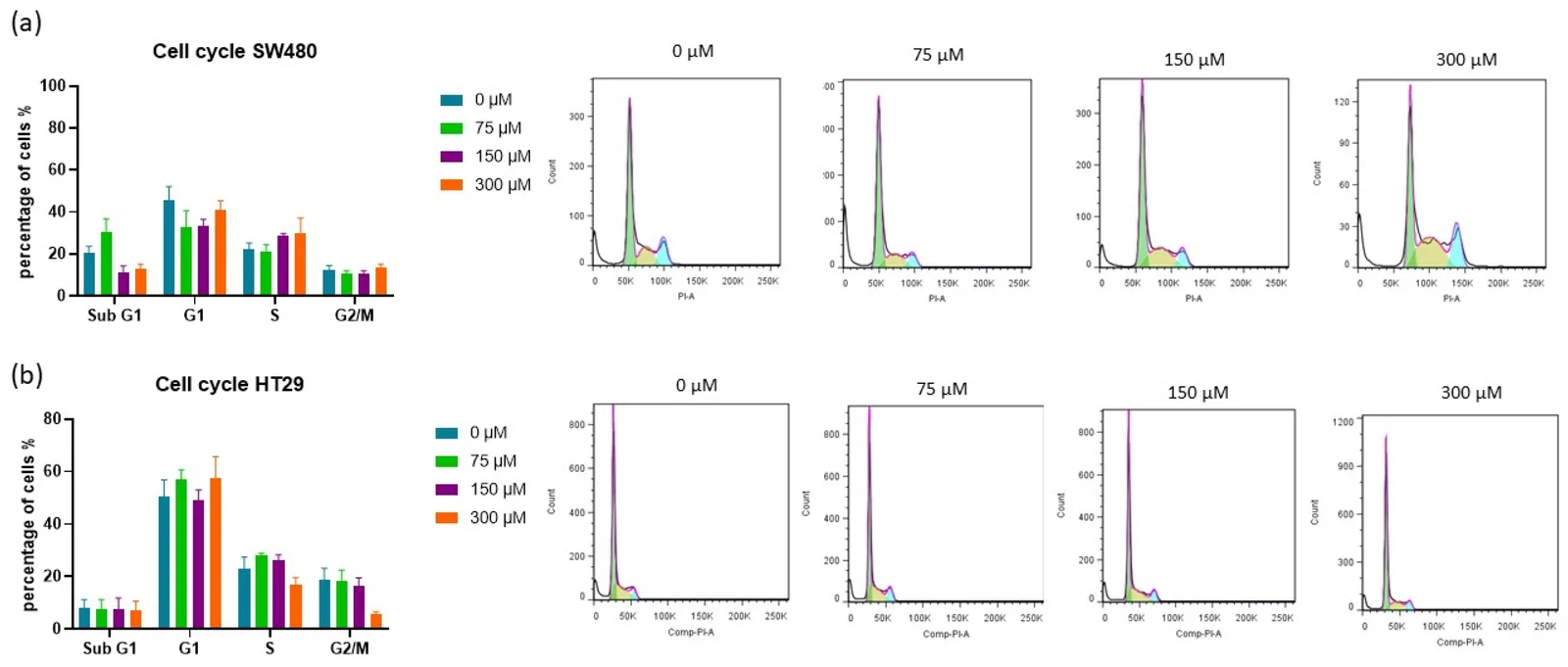
Effect of *n*-butyl chlorogenate on cell cycle distribution in CRC cells. Cell cycle distribution was analyzed by flow cytometry following PI staining of DNA content after treatment with *n*-butyl chlorogenate (75, 150, 300 µM) in (**a**) SW480 and (**b**) HT-29 cells. Bar graphs show the percentage of cells in the sub-G1, G1, S, and G2/M phases in both untreated and treated groups. Data are presented as the mean ± SEM from three independent experiments. Representative DNA content histograms are shown on the right.

To further investigate the cellular effects of *n*-butyl chlorogenate, cell cycle distribution was analyzed by flow cytometry to determine whether treatment affected cell proliferation. DNA content was assessed by PI staining, enabling quantification of cell populations across cell cycle phases. In SW480 cells (Figure 7a), treatment with *n*-butyl chlorogenate at 75 µM resulted in a slight, non-significant increase in the sub-G1 population, accompanied by a decrease in the proportion of cells in the G1 phase, whereas no appreciable changes were observed in the S or G2/M phases.

Higher concentrations (150 and 300 µM) did not produce detectable alterations in cell cycle distribution. Similarly, HT-29 cells (Figure 7b) exhibited no significant alterations in cell cycle phase distribution following treatment with *n*-butyl chlorogenate. Overall, these results indicate that *n*-butyl chlorogenate does not affect cell cycle progression in either SW480 or HT-29 cells under the experimental conditions evaluated.

## 3. Discussion

The present study demonstrates that esterification enhances the biological activity of CGA against CRC cells. Among the evaluated derivatives, *n*-butyl chlorogenate exhibited the strongest cytotoxic effect, particularly in SW480 cells. In contrast, only limited effects were observed in non-tumoral NCM460 cells within the tested concentration range (0–1000 μM). In contrast, HT-29 cells showed lower sensitivity under the same experimental conditions, suggesting differential responses among CRC cell lines.

Previous studies from our research group have reported that native CGA requires concentrations between 1000 and 2000 μM to induce significant antiproliferative effects in SW480 and HT-29 cells. Since these findings were previously established under comparable experimental conditions, native CGA was not included as a direct experimental control in the present study [9,20].

The molecular docking results obtained in the present study are consistent with our previous report demonstrating that CGA exhibits higher predicted affinity for both the union site and allosteric binding sites of β-catenin than the reference inhibitors iCRT14 and C2, mainly through an extensive hydrogen-bond network that supports its potential modulation of the Wnt/β-catenin pathway [20].

Consistent with those findings, native chlorogenic acid exhibited the strongest predicted affinity among all evaluated compounds in the present study. Although esterification progressively reduced binding affinity, all CGA esters largely preserved the parent molecule’s binding orientation at both β-catenin subsites, indicating that structural modification did not abolish molecular recognition of the target. Instead, the decrease in affinity appeared to result from the gradual loss of stabilizing interactions, particularly hydrogen bonds and π-stacking contacts, as the alkyl chain length increased.

Interestingly, these docking predictions did not correlate directly with the biological activity observed in vitro. Despite exhibiting the weakest predicted affinity for β-catenin, *n*-butyl chlorogenate showed the highest cytotoxic activity against colorectal cancer cells. This apparent discrepancy suggests that molecular recognition of β-catenin alone is unlikely to explain the improved biological activity of the ester derivatives. Instead, physicochemical properties introduced by esterification may play an important role. In silico characterization revealed a progressive increase in lipophilicity, with Consensus LogP values increasing from −0.71 for chlorogenic acid to 0.67 for *n*-butyl chlorogenate. Increased lipophilicity has been associated with enhanced membrane permeability, potentially facilitating greater intracellular accumulation and increasing the effective concentration available to interact with intracellular molecular targets despite the lower predicted binding affinity. Therefore, the enhanced cytotoxic activity of the longer-chain esters is more likely attributable to improved cellular uptake than to stronger interaction with β-catenin.

This interpretation is also supported by recent computational studies showing that CGA consistently exhibits favorable interactions with both β-catenin binding sites, reinforcing β-catenin as a plausible molecular target while highlighting that docking affinity alone cannot predict the overall biological activity of structurally modified derivatives [6]. However, despite this promising biological profile, the physicochemical properties of native CGA may limit their pharmacological performance [20,23].

In the present study, *n*-butyl chlorogenate reached its IC_50_ within the tested concentration range (0–1000 μM), suggesting that esterification, particularly the introduction of a butyl group, may enhance the cytotoxic potency of CGA. Moreover, a progressive increase in cytotoxic activity was observed as the alkyl chain length increased from methyl to *n*-butyl esters, supporting a structure-activity relationship associated with increased lipophilicity.

The in silico characterization further supported this trend. Esterification progressively increased Consensus LogP values while reducing predicted aqueous solubility, indicating substantial modifications in the physicochemical profile of the ester derivatives. In addition, all esters complied with Lipinski’s rule of five, whereas none fulfilled Veber’s rule because their predicted topological polar surface area values exceeded the recommended threshold. These findings are consistent with previous in silico studies reporting that structural modification of CGA improved predicted physicochemical properties and drug-likeness profiles while preserving characteristics relevant for drug development [24,25,26]. Altogether, these results suggest that although esterification represents a promising strategy to enhance the physicochemical profile of CGA, additional optimization may still be required to further improve properties associated with oral drug development; whether the increased lipophilicity enhances membrane permeability or intracellular accumulation requires direct experimental confirmation.

Due to their affinity for lipid environments, lipophilic molecules can localize within mitochondrial membranes, where they may influence redox processes [27]. In the present study, *n*-butyl chlorogenate induced significant changes in mitochondrial oxidative status in SW480 cells at concentrations substantially lower than those previously reported for native CGA. Whereas the oxidative effects induced by CGA have generally been described at concentrations above 1000 μM [20], and changes in MitoTracker Red CM-H2Xros (M7513, Invitrogen, Carlsbad, CA, USA) fluorescence were detected in the present study at concentrations ranging from 75 to 300 μM. These findings suggest that esterification may enhance the compound’s ability to alter mitochondrial oxidative status; however, further studies are required to determine whether these changes are directly associated with increased mitochondrial ROS production.

Mitochondrial oxidative stress has been widely recognized as an important mediator of cell death signaling. In the present study, SW480 cells exposed to *n*-butyl chlorogenate exhibited increased phosphatidylserine externalization, alterations in membrane integrity, and increased cellular complexity, all of which are compatible with early apoptotic features. Although these findings may be associated with the observed changes in mitochondrial oxidative status, no significant activation of Caspase-3/7 was detected at the time point evaluated.

The absence of detectable Caspase-3/7 activation does not necessarily exclude the involvement of apoptotic signaling pathways. Apoptosis is a dynamic process in which caspase activation may be transient and vary according to cell type and treatment conditions.

In the present study, Caspase-3/7 activity was measured 24 h after exposure, a time point frequently used to evaluate apoptotic responses in CRC cell lines, including HCT116, LoVo, HT-29, SW620 and SW480 [28,29,30]. Nevertheless, previous studies in CRC models have reported caspase activation at different time points, ranging from a few hours after treatment to 48 h of exposure [31,32]. Importantly, caspase activation is often transient and closely linked to the progression of apoptosis. As cells move from early to later stages of apoptosis, detectable caspase activity may decline even though apoptotic cell death continues. For this reason, measurements obtained at a single time point provide only a snapshot of this dynamic biological process [33].

Therefore, the increase in Annexin V positivity together with the observed changes in mitochondrial oxidative status may be consistent with either an early apoptotic response or the involvement of caspase-independent cell death mechanisms. Mitochondrial oxidative stress can promote outer membrane permeabilization and the release of apoptogenic factors such as apoptosis-inducing factor (AIF), leading to cell death independently of classical executioner caspases. Similar caspase-independent cell deaths have been reported for other cytotoxic compounds in different cancer models [34,35,36].

The greater sensitivity of SW480 cells compared with HT-29 cells may be explained by intrinsic molecular differences between these CRC cell lines. Previous studies have shown that the uptake and biological activity are influenced by cell-specific characteristics, including membrane composition, metabolic profile, and antioxidant capacity [37,38]. HT-29 cells have also been reported to possess mechanisms associated with reduced intracellular accumulation of therapeutic agents. These include increased expression of drug efflux transporters, such as the breast cancer resistance protein (BCRP), and variable expression of UDP-glucuronosyltransferases (UGTs), enzymes that catalyze glucuronidation reactions and facilitate the elimination of xenobiotics, including phenolic compounds [39,40]. Together, these characteristics may contribute to the lower sensitivity of HT-29 cells observed in the present study.

Additionally, differences in signaling pathways may also influence responsiveness. Dysregulation of the Wnt/β-catenin pathway is a hallmark of CRC and plays a central role in tumor proliferation and survival. Previous studies have demonstrated that CGA can modulate Wnt/β-catenin signaling in SW480 cells, and that co-treatment of CGA with 5-fluorouracil regulates the expression of genes such as CTNNB1, TCF7L2, JUN, and CCND1 more effectively in SW480 than in HT-29 cells [9,41]. Furthermore, monocarboxylate transporters (MCTs), especially MCT1, are critical to lactate metabolism and tumor cell proliferation, as they are involved in the Warburg effect and have also been associated with the transport of polyphenols and their metabolites [42,43]. Likewise, CGA-mediated downregulation of SLC16A1, which encodes the monocarboxylate transporter MCT1, has been reported predominantly in SW480 cells [41]. These observations suggest that the molecular context of SW480 cells may favor responsiveness to CGA and its derivatives, potentially contributing to the enhanced sensitivity observed for *n*-butyl chlorogenate.

Although CGA has been extensively investigated for its anticancer properties, relatively few studies have explored the biological activity of its ester derivatives. Most previous reports involving *n*-butyl chlorogenate have focused on antioxidant properties and physicochemical characterization rather than cytotoxic activity [44,45,46,47]. Therefore, the present study provides novel evidence supporting the antitumoral potential of this derivative in CRC models.

The enhanced activity observed for *n*-butyl chlorogenate is consistent with the principle that moderate increases in lipophilicity may improve membrane permeability and intracellular accessibility [48]. Lipophilized phenolic compounds have been reported to exhibit greater affinity for biological membranes and lipid interfaces, which may facilitate accumulation in lipid-rich organelles such as mitochondria [11,12,49,50,51,52]. Therefore, the enhanced cytotoxic activity observed for *n*-butyl chlorogenate may be associated with its increased lipophilicity. Likewise, the changes in mitochondrial oxidative status observed in SW480 cells may reflect an early cellular response associated with its cytotoxic effect.

The present findings should be interpreted within the context of the known limitations of native CGA, which has been reported to exhibit low oral bioavailability and to require relatively high concentrations to produce biological effects in vitro [53]. In the present study, *n*-butyl chlorogenate exhibited greater cytotoxic activity than the other synthesized derivatives, reaching an IC_50_ within the tested concentration range. These findings suggest that structural modification of CGA through esterification can influence its physicochemical properties and biological activity. However, the cytotoxic effects were still observed at relatively high micromolar concentrations, indicating that further structural optimization will be required to improve cytotoxic potency. Therefore, the present study should be considered as a proof of concept supporting esterification as a strategy for optimizing chlorogenic acid derivatives, rather than as evidence of immediate therapeutic applicability.

Nevertheless, some limitations should be acknowledged. First, the study was conducted in silico and in vitro; therefore, the observed effects may not fully reflect the complexity of the tumor microenvironment in vivo. In addition, two-dimensional cell culture models do not reproduce the cellular interactions, nutrient gradients, and drug penetration characteristics present in three-dimensional tumor systems.

Second, native CGA was not included as a direct experimental control in the present study because our research group had already characterized its biological activity in the same laboratory using the same colorectal cancer cell lines and comparable experimental conditions, and these findings have been previously published [8,20,54]. Therefore, the experiments with native CGA were not repeated, and the comparisons discussed in this manuscript are based on these previously published data rather than on side-by-side experiments performed as part of the present study. Consequently, direct experimental comparisons between native CGA and its ester derivatives cannot be established. However, the literature-based comparisons rely on data generated by our group under comparable experimental conditions.

Third, the in silico characterization was based on predictive physicochemical descriptors. Although these computational tools are valuable for estimating drug-like properties, they provide theoretical predictions that require experimental validation and should not be interpreted as direct evidence of pharmacokinetic behavior. Molecular docking also has limitations; for instance, it directly presents the ligand to the target site. Without taking into account pharmacokinetic and pharmacodynamic processes or the cellular environment. Therefore, this methodology is not enough to confirm interaction and pathway modulation.

Finally, although increased lipophilicity was associated with enhanced biological activity, membrane permeability, cellular uptake, and intracellular accumulation were not directly evaluated. Therefore, the proposed relationship between these properties remains hypothetical and requires further experimental confirmation. In addition, although esterification improved the cytotoxic activity of the synthesized ester derivatives, the biological effects were observed at relatively high micromolar concentrations, indicating that further structural optimization will be necessary to improve potency before considering progression toward preclinical in vivo evaluation. Future studies incorporating permeability assays, direct cellular uptake analyses, three-dimensional culture models, and in vivo validation will be required to further characterize and optimize the pharmacological potential of CGA ester derivatives for CRC.

## 4. Materials and Methods

### 4.1. Synthesis of CGA Alkyl Esters

Four chlorogenic acid esters were synthesized, purified, and characterized. Methyl, ethyl, *n*-propyl, and *n*-butyl chlorogenates were obtained by Fischer esterification (see Table 4 for the alkyl substituent groups of the synthesized esters). CGA was dissolved in an excess of the corresponding alcohol (methanol, ethanol, *n*-propanol, or *n*-butanol). Concentrated H_2_SO_4_ was added as a catalyst, and the reaction mixtures were refluxed for 1–3 h. Reaction progress was monitored by thin-layer chromatography (TLC) on silica gel 60 F254 plates until complete consumption of CGA. Subsequently, the reaction mixtures were neutralized and extracted with ethyl acetate. The organic phase was filtered over anhydrous sodium sulfate and evaporated under reduced pressure using a rotary evaporator to obtain the crude product.

The crude products were purified by column chromatography on silica gel 60 (230–400 mesh) using a chloroform:ethyl acetate:formic acid (5:4:1) solvent system, followed by recrystallization from ethanol. When necessary, fractions containing the target compound were further purified by semipreparative high-performance liquid chromatography (HPLC) using an Agilent Eclipse XDB-C18 column (5 µm, 9.4 × 250 mm; Agilent Technologies, Santa Clara, CA, USA). Compound purity was assessed by analytical HPLC using a KNAUER Eurospher II 100-5 C18 column (4.6 × 250 mm) (KNAUER Wissenschaftliche Geräte GmbH, Berlin, Germany), and a gradient mobile phase ranging from 100% H_2_O + 0.1% formic acid to 100% (acetonitrile + 0.1% formic acid).

**Figure 8:**
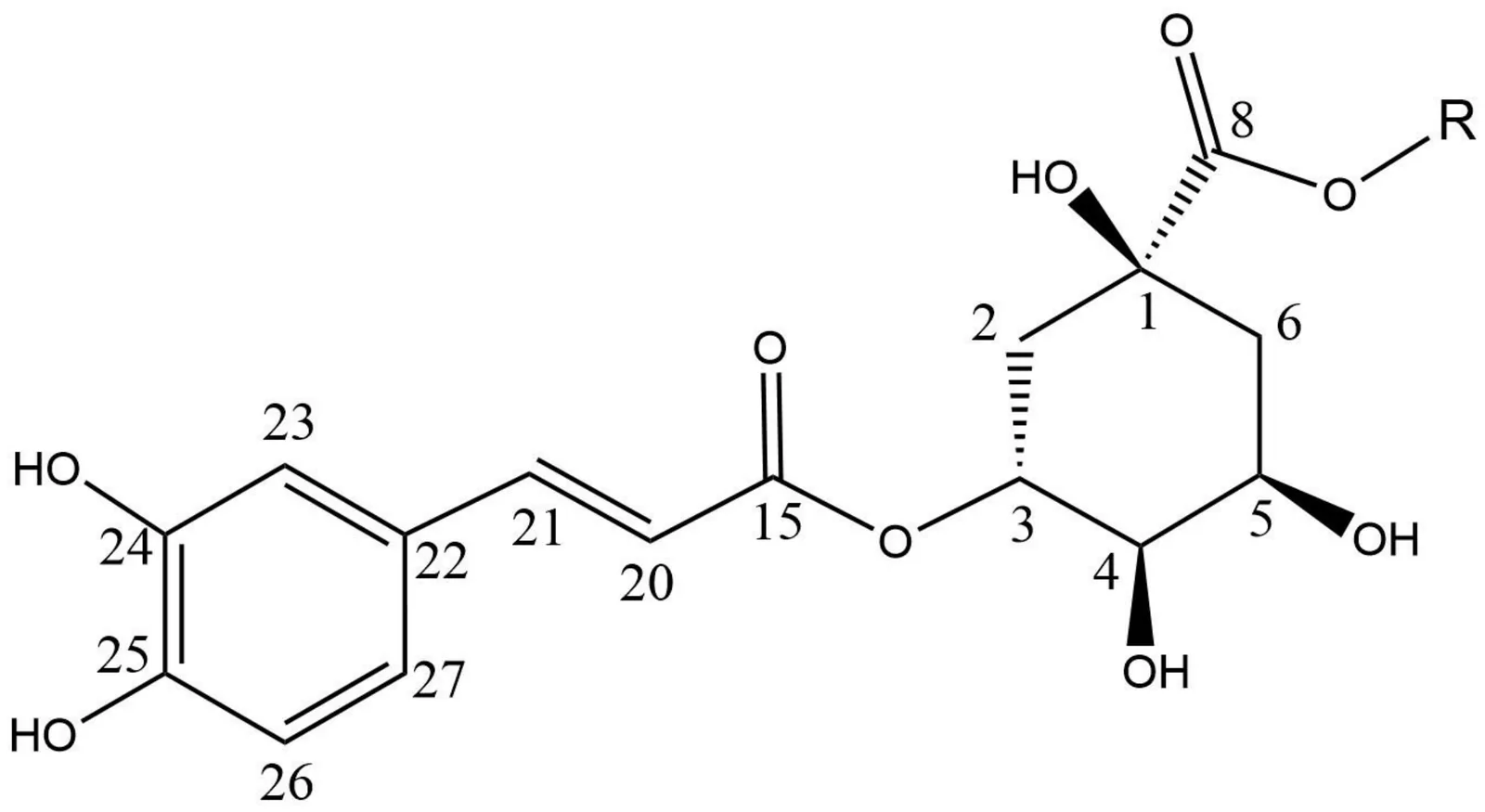
Synthesized chlorogenic acid esters and their chemical structures.

NMR characterization was performed using a Bruker Ascend HD III BioSpin 600 MHz spectrometer (Bruker, Billerica, MA, USA) equipped with a 5 mm TXI 600S3 H-C/N-D-05-Z probe (see Figure 8 for atom). All compounds were dissolved in deuterated methanol, and ^1^H NMR spectra were obtained for each synthesized ester; For *n*-butyl chlorogenate, ^13^C and HSQC NMR spectra were also acquired.

For the biological assays, stock solutions of each purified chlorogenic acid ester were prepared in absolute ethanol and diluted in complete culture medium immediately before use to obtain the desired working concentrations. The final ethanol concentration in the culture medium did not exceed 0.3% (*v*/*v*). Vehicle-treated cells containing the same final ethanol concentration were included as solvent controls throughout the biological assays.

### 4.2. In Silico Physicochemical Analysis

In silico physicochemical characterization was performed using the SwissADME web tool (http://www.swissadme.ch/ (accessed on 23 August 2025)). Drug-likeness was evaluated according to Lipinski’s rule of five (molecular weight, MLOGP, hydrogen-bond acceptors, and hydrogen-bond donors) and Veber’s rule (topological polar surface area and rotatable bonds). In addition, lipophilicity and aqueous solubility were assessed using predicted Consensus LogP and LogS (ESOL) values, respectively.

### 4.3. Cell Culture

The human colorectal adenocarcinoma cell lines SW480 (ATCC^®^ CCL-228; ATCC, Manassas, VA, USA) and HT-29 (ATCC^®^ HTB-38^TM^, ATCC, Manassas, VA, USA), together with the non-tumoral human colon cell line NCM460 (Cytion catalog No. 305430, Cytion GmbH, Berlin, Germany), were used in this study. Cells were cultured at 37 °C in Dulbecco’s Modified Eagle Medium (DMEM) supplemented with 5% fetal bovine serum (FBS), or Roswell Park Memorial Institute medium RPMI supplemented with 10% FBS, respectively, and 100 μg/mL penicillin and 100 μg/mL streptomycin. Cells were maintained at 37 °C in a humidified atmosphere containing 5% CO_2_. All experiments employed exponentially growing cultures. Subcultures were maintained by cell dissociation with trypsin-EDTA (0.1–0.25 mM) for 4–6 min, followed by mechanical detachment. Trypsin activity was neutralized by adding 5 mL of complete culture medium. The cell suspension was centrifuged at 3500 rpm for 4 min; the supernatant was discarded, and the pellet was resuspended in complete medium. Cells were subcultured twice a week or before reaching confluence.

### 4.4. Cytotoxicity Assay (MTT)

An initial analysis was performed with four CGA ester derivatives (methyl, ethyl, *n*-propyl, and *n*-butyl chlorogenates). Concentrations tested were 1, 10, 100, and 1000 μM. The concentration range (1–1000 μM) was selected to perform an initial dose–response screening over a broad concentration interval, in accordance with the recommendations of ISO 10993-5 for in vitro cytotoxicity testing, which recommends testing four serial concentrations covering a broad concentration range, and was also based on concentrations previously reported for CGA in CRC studies.

Cells were seeded in triplicate in 96-well microplates containing 100 μL of culture medium. After 24 h, compounds were added, and following 24 h of exposure, 100 μL of MTT (0.5 mg/mL) was added. Cultures were re-incubated at 37 °C for 2 h, followed by the addition of acidic isopropanol (100 μL) for 10 min to solubilize the formazan crystals produced by viable cells. Absorbance was measured at 570 nm using the Varioskan multimode microplate reader (Thermo Scientific, Waltham, MA, USA). Untreated cells were considered 100% viable. The ester with the best response was selected, and the working concentration was determined based on the IC_50_ value. IC_50_ values were estimated by nonlinear regression using the Dose–Response (Inhibition) model implemented in GraphPad Prism (version 8.0, GraphPad Software, San Diego, CA, USA). The model was fitted to the experimental concentration–response data to estimate the IC_50_ for each compound.

### 4.5. Cell Death Assays

#### 4.5.1. DiOC and Propidium Iodide (PI) Assay

Cells were seeded and treated in 6-well plates with the selected concentration of the CGA ester, using untreated cells as the control. After 24 h, cells were washed three times with PBS, trypsinized, and processed using DiOC6 (D273, Molecular Probes, Eugene, OR, USA) with Propidium Iodide (PI, P4170, Sigma-Aldrich, St. Louis, MO, USA) for Flow Cytometry, following manufacturer instructions. A total of 10,000 events were analyzed by flow cytometry using a BD LSRFortessa cell analyzer (BD Biosciences, San Jose, CA, USA). Populations positive for Sytox (Thermo Fisher Scientific, Waltham, MA, USA), positive for Annexin V-PE (BD Biosciences, San Jose, CA, USA), or negative for both markers were quantified using FlowJo software version 7.6.2 (BD Life Sciences, Ashland, OR, USA).

#### 4.5.2. MitoTracker Assay

Cells were seeded in 6-well plates and exposed to the selected CGA ester concentration, using untreated cells as the control. After 24 h, cells were washed three times with PBS, trypsinized, and processed with MitoTracker Red CM-H2Xros (M7513, Invitrogen, Carlsbad, CA, USA) for Flow Cytometry, following manufacturer instructions. A total of 10,000 events were analyzed by flow cytometry using a BD LSRFortessa cell analyzer. Populations positive for Sytox, positive for Annexin V-PE, or negative for both markers were quantified using FlowJo software.

#### 4.5.3. Annexin V and Sytox Green Staining

Cells were seeded in 6-well plates and exposed to the selected CGA ester concentration, using untreated cells as the control. After 24 h, cells were washed three times with PBS, trypsinized, and processed using the Dead Cell Apoptosis Kit with Annexin V for Flow Cytometry (Invitrogen, V35112), following manufacturer instructions. A total of 10,000 events were analyzed by flow cytometry using a BD LSRFortessa cell analyzer. Populations positive for Sytox, positive for Annexin V-PE, or negative for both markers were quantified using FlowJo software.

#### 4.5.4. Caspase-3/7 Staining

Cells were seeded in 6-well plates and allowed to adhere for 24 h. Experiments were performed using the selected CGA ester concentration, using non-exposed cells as the control. After 24 h of exposure, culture media and cells were collected and centrifuged. CellEvent™ Caspase-3/7 Green Detection Reagent (1 µL; Thermo Fisher Scientific Waltham, MA, USA) was added to each sample and incubated for 30 min. During the last 5 min, 1 µL of SYTOX™ AADvanced (Thermo Fisher Scientific, Waltham, MA, USA) dead cell stain was added. Samples were analyzed by flow cytometry (BD LSRFortessa), acquiring 10,000 events.

### 4.6. Cell Proliferation Assays

#### Cell Cycle Assay

Cells were seeded into 6-well plates and exposed to the selected concentrations of CGA esters, using non-exposed cells as the control. After 24 h, cells were washed three times with PBS, trypsinized, and transferred into 15 mL Falcon tubes. Cells were fixed by adding 200 µL of cold 70% ethanol dropwise while gently vortexing, followed by incubation at 4 °C for 24 h. For propidium iodide (PI) staining, ethanol was removed, and cells were washed three times with PBS to eliminate residual fixative. PI staining was then performed according to the manufacturer’s instructions. A total of 10,000 events per sample were acquired using a BD LSRFortessa™ flow cytometer. Cell cycle distribution was quantified using FlowJo™ software.

### 4.7. Molecular Docking

Chlorogenic acid anion and the ester derivatives were built and prepared using GaussView 6 [55] and were geometry-optimized in GAUSSIAN 16W [56] at the B3LYP-6-31++G(d,p) level of approximation. All ligands were converted to PDBQT format using AutoDock Tools (ADT) version 1.5.7 (The Scripps Research Institute, La Jolla, CA, USA). [57].

The structure of β-catenin (PDB code 2GL7) was prepared using UCSF ChimeraX version 1.12, San Francisco City, CA, USA [58] and ADT; polar hydrogen atoms were automatically added, and AD4-type atoms and Gasteiger charges were assigned.

The docking parameters were established according to the literature. β-catenin has two known binding sites: the union site (β-catenin US), located at the most crucial interaction between the residues Asp16 from TCF4 and Lys435 and His470; and the allosteric site (β-catenin AS), which included the residues Pro521, Arg528, and Asp583 [20].

Molecular docking was carried out in AutoDock Vina version 1.2.3 (The Scripps Research Institute, La Jolla, CA, USA) using the following grid parameters: union site (center: x = 11.527, y = 22.308, z = 62.347; Size: 17 Å^3^), allosteric site (center: x = 2.805, y = 14.864, z = 79.543; Size: 17 Å^3^) [59]. The pose with the best affinity for each site was selected, and a visual inspection of the interactions was performed using Discovery Studio Visualizer (BIOVIA) and UCSF Chimera X.

### 4.8. Statistical Analysis

Descriptive analyses were first performed to visualize data distribution in terms of frequency, central tendency, and dispersion. Data normality was assessed using the Shapiro–Wilk test. For the cytotoxicity assays, differences in cell viability were analyzed using two-way analysis of variance (ANOVA), considering treatment concentration and exposure time as the two factors, followed by Tukey’s multiple comparison test. For the remaining biological assays, one-way ANOVA followed by Tukey’s multiple comparison test was applied to normally distributed data, whereas the Kruskal–Wallis test followed by Dunn’s multiple comparison test was used for non-normally distributed data. All experiments were performed using three independent biological replicates, each including three technical replicates. Statistical significance was established at *p* < 0.05. All statistical analyses were performed using GraphPad Prism version 8.0 (GraphPad Software, San Diego, CA, USA).

## 5. Conclusions

Esterification of chlorogenic acid enhanced the cytotoxic activity against colorectal cancer cells, mostly in SW480 cells, while only limited effects were observed in non-tumoral NCM460 cells, within the tested concentration range. The progressive increase in alkyl chain length, probably accompanied by increased lipophilicity, was associated with enhanced cytotoxic activity. In this context, *n*-butyl chlorogenate induced changes in mitochondrial oxidative status together with apoptosis-associated cellular changes, suggesting that oxidative stress may contribute to its cytotoxic effects.

In silico physicochemical characterization indicated that esterification increased lipophilicity while reducing predicted aqueous solubility. Although the ester derivatives fulfilled Lipinski’s rule-of-five, none satisfied Veber’s rule because of their high predicted TPSA, suggesting that further structural optimization may be required to improve physicochemical properties relevant to drug development.

The higher biological activity observed at concentrations lower than those previously reported for native CGA suggests that esterification enhances the cytotoxic activity. This effect may be related to the increased lipophilicity of the esters; however, additional studies are required to determine whether enhanced cellular uptake and/or permeability are responsible.

Moreover, the differential responses observed between SW480 cells and HT-29 cells highlight the importance of cellular context in determining cellular sensitivity to treatment. Overall, these findings demonstrate that rational chemical modification through esterification can modify the physicochemical profile and enhance the cytotoxic activity of CGA derivatives in CRC cell lines. Although the chlorogenic acid esters exhibited enhanced cytotoxic activity in CRC cells, further structural optimization will be necessary before evaluating their potential for in vivo CRC studies.

## Figures and Tables

**Table 1:** Predicted physicochemical and drug-like properties of CGA and its ester derivatives.

	MW	Consensus LogP	HBA	HBD	TPSA	Log S (ESOL)	Rotatable Bonds	Lipinski’s Rules	Veber’s Rule
CGA	354.31 g/mol	−0.71	9	6	164.75 ${Å}^{2}$	−1.62	5	Yes; 1 violation: HBD > 5	No; 1 violation: TPSA > 140
methyl chlorogenate	368.34 g/mol	−0.41	9	5	153.75 ${Å}^{2}$	−1.84	6	Yes; 0 violations	No; 1 violation: TPSA > 140
ethyl chlorogenate	382.36 g/mol	−0.06	9	5	153.75 ${Å}^{2}$	−2.08	7	Yes; 0 violations	No; 1 violation: TPSA > 140
*n*-propyl chlorogenate	396.39 g/mol	0.33	9	5	153.75 ${Å}^{2}$	−2.43	8	Yes; 0 violations	No; 1 violation: TPSA > 140
*n*-butyl chlorogenate	410.42 g/mol	0.67	9	5	153.75 ${Å}^{2}$	−2.67	9	Yes; 0 violations	No; 1 violation: TPSA > 140

**Lipinski’s rule of five criteria**: molecular weight (MW) ≤ 500 g/mol, MLOGP ≤ 5, hydrogen-bond acceptors (HBA) ≤ 10, and hydrogen-bond donors (HBD) ≤ 5. **Veber’s rule criteria**: Rotatable bonds ≤ 10 and Topological Polar Surface Area (TPSA) ≤ 140 ${Å}^{2}$. Physicochemical properties and drug-likeness descriptors were predicted using the SwissADME web tool. Molecular weight (MW), H-bond acceptors (HBA), and H-bond donors (HBD).

**Table 2:** Affinity and protein-ligand interactions of chlorogenic acid and ester derivatives with β-catenin at the union (US) and the allosteric (AS) sites.

Protein/Subsite	Compound	Vina Score [kcal/mol]	Protein-Ligand Interactions		
			Hydrogen Bonds	π Interactions	Hydrophobic Interactions
**β-catenin/AS**	Chlorogenic acid	−5.5	ARG587 ASP583 HIS585 PRO521 HIS524 ALA525 GLN623		ALA522 ARG528 ARG582 VAL584
	Methyl chlorogenate	−5.4	ARG587 ASP583		GLN623 ARG582 VAL584 HIS585 ARG528 ALA525 PRO521 HIS524 ALA522
	Ethyl chlorogenate	−5.0	LEU519 GLN623 ASP583		CYS520 ALA518 GLU620 VAL584 PRO521
	*n*-propyl chlorogenate	−5.0	LEU519 VAL584 ARG587 ARG582		CYS520 ALA518 GLU620 GLN623 ASP583 PRO521
	*n*-butyl chlorogenate	−5.0	ARG587		ALA518 LEU519 CYS520 HIS524 ARG582 ASP583 VAL584
**β-catenin/US**	Chlorogenic acid	−6.5	ASN430 LYS435 SER473 ARG474 GLY512 ASN516	HIS470	THR428 LYS508 GLU571 ARG469
	Methyl chlorogenate	−6.3	ASN430 LYS435 ARG474 ARG515 ASN516	HIS470	ARG469 SER473 LYS508 GLY512 CYS429
	Ethyl chlorogenate	−6.3	ASN430 LYS435 ARG474 GLY512 ARG515 ASN516	HIS470	THR428 ASN431 ARG469 SER473 LYS508 GLU571 CYS429
	*n*-propyl chlorogenate	−6.0	LYS435 ARG474 ARG515		THR428 ASN430 ARG469 SER473 LYS508 GLY512 ASN516 GLU571
	*n*-butyl chlorogenate	−5.6	LYS435 ARG474 GLY512		THR428 ASN431 SER473 ASN516 LEU513 ARG515 ARG469 CYS429 LEU519

**Table 3:** IC_50_ values of chlorogenic acid esters in colorectal cancer cell lines.

	CGA Esters	24 h	48 h
SW480	Methyl Chlorogenate	ND *	3561
	Ethyl Chlorogenate	7348	2653
	*n*-propyl Chlorogenate	3148	1141
	*n*-butyl Chlorogenate	658.9	341.1
HT-29	Methyl Chlorogenate	ND *	3674
	Ethyl Chlorogenate	ND *	2498
	*n*-propyl Chlorogenate	8162	1696
	*n*-butyl Chlorogenate	4168	779

IC_50_ values are expressed in μM. ND *: IC_50_ values could not be determined within the tested concentration range (0–1000 μM). IC_50_ values were estimated by nonlinear regression analysis using a four-parameter logistic model in GraphPad Prism (version 8, GraphPad Software, San Diego, CA, USA).

**Table 4:** Substituent groups (R) of chlorogenic acid esters.

Compound	R
Methyl chlorogenate	–CH_3_
Ethyl chlorogenate	–CH_2_–CH_3_
*n*-propyl chlorogenate	–CH_2_–CH_2_–CH_3_
*n*-butyl chlorogenate	–CH_2_–(CH_2_)_2_–CH_3_

R represents the alkyl substituent esterified to chlorogenic acid, defining the ester derivatives evaluated in this study.

## Data Availability

The data are not available in a public repository; however, the supporting data are available from the corresponding laboratory upon reasonable request.

## Supplementary Materials

The following supporting information can be downloaded at: https://www.mdpi.com/article/10.3390/molecules31172952/s1; Figure S1: NMR spectra of M1_ Methyl chlorogenate ester, Figure S2. NMR spectra of M2 Ethyl chlorogenate ester, Figure S3. NMR spectra of M3 Propyl chlorogenate ester, Figure S4. NMR spectra of M4 Butyl chlorogenate ester.
