# Supplementary material for: Improved Lipophilicity Is Associated with the Cytotoxic Activity of Chlorogenic Acid Esters in In Vitro Colorectal Cancer Models
Source: Molecules. 2026 Aug 23;31(17):2952. doi: 10.3390/molecules31172952 (PMC13567004; doi:10.3390/molecules31172952)
Supplement: Supplementary file 1 [file molecules-31-02952-s001.zip › molecules-4429548-supplementary.pdf]

Supplementary material- NMR spectra of CGA esters

M1_ Methyl chlorogenate

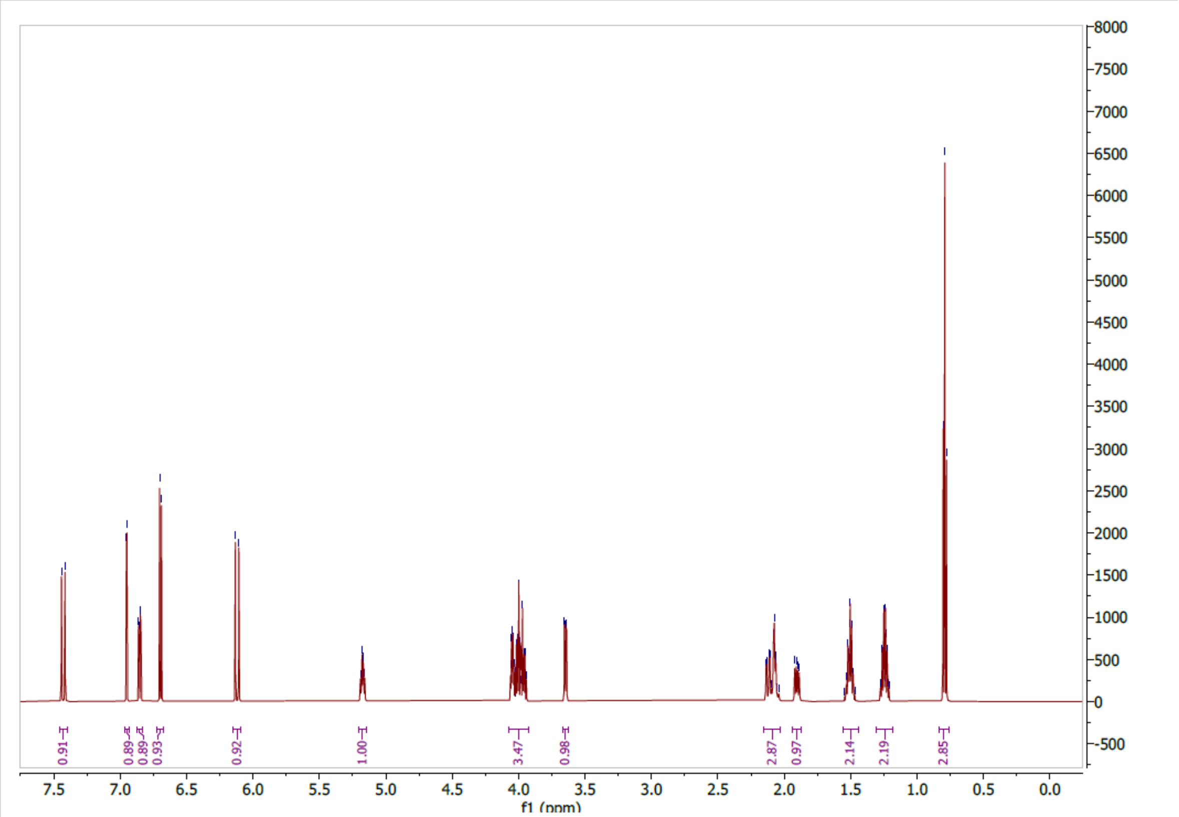

Figure S1. NMR spectra of M1_ Methyl chlorogenate ester.

M2_ Ethyl chlorogenate

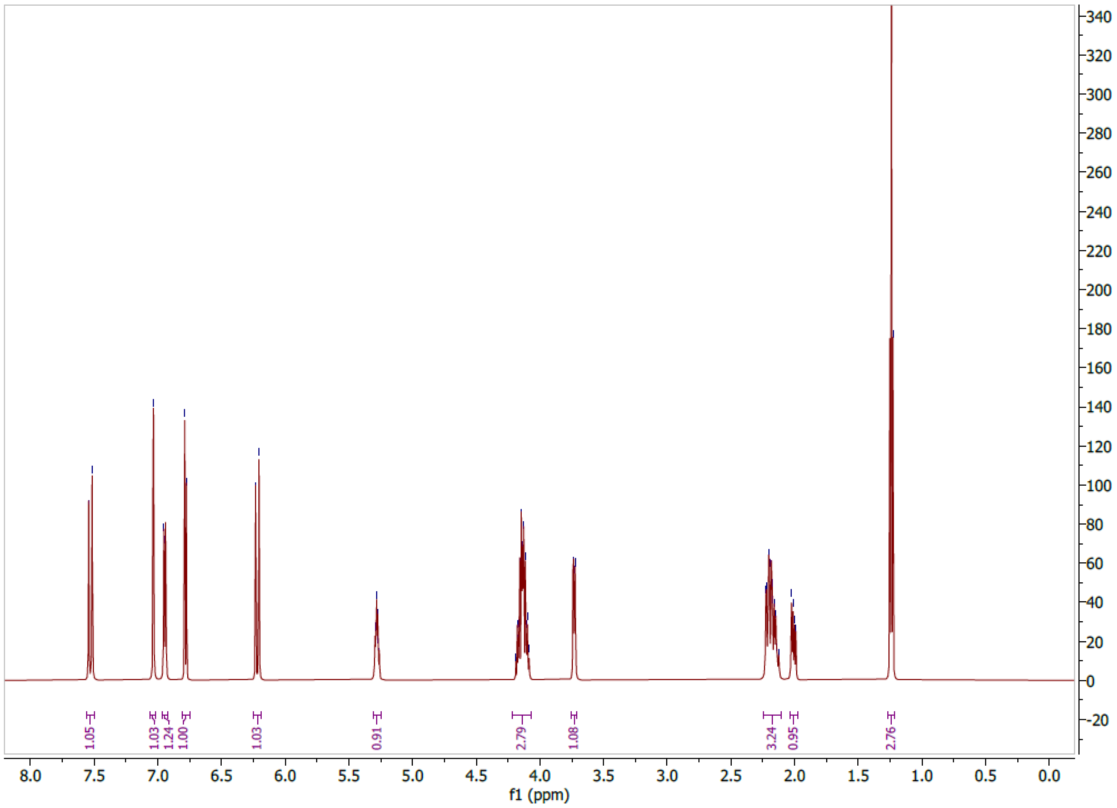

Figure S2. NMR spectra of M2_ Ethyl chlorogenate ester.

M3_ Propyl chlorogenate

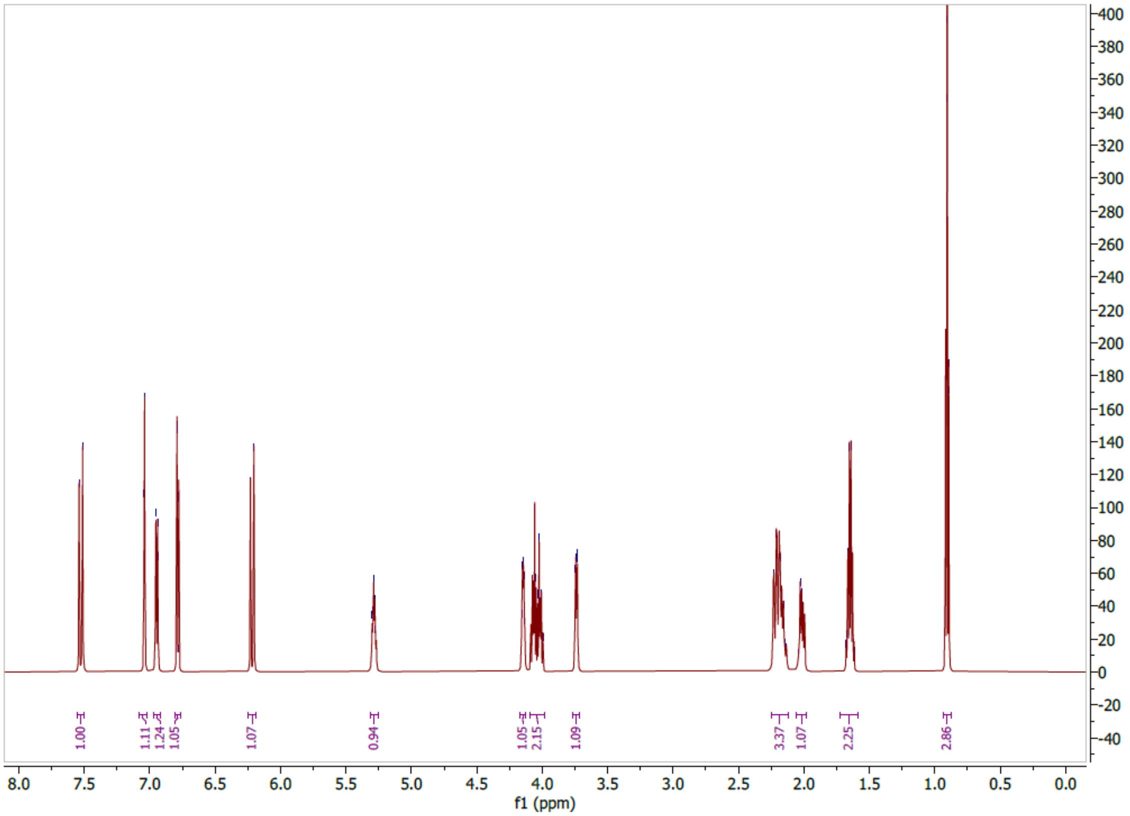

Figure S3. NMR spectra of M3_ Propyl chlorogenate ester.

M4_ Butyl chlorogenate

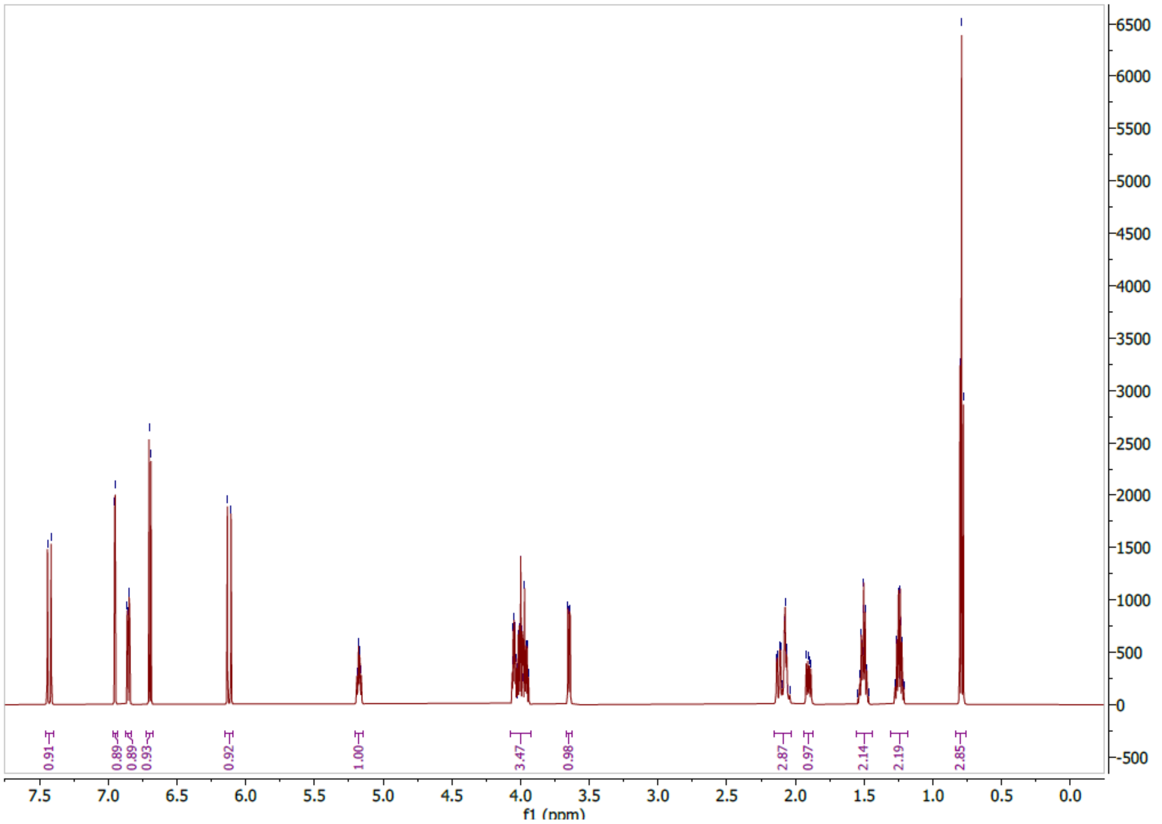

Figure S4. NMR spectra of M4_ Butyl chlorogenate ester.
